# Immune-mediated febrile response in female rats: Role of central hypothalamic mediators

**DOI:** 10.1038/s41598-020-61210-z

**Published:** 2020-03-05

**Authors:** Haissa Oliveira Brito, Débora Radulski, Daniel Björk Wilhelms, Andrea Stojakovic, Luciane Maria Oliveira Brito, Rui Miguel Gil da Costa, Edvaldo Trindade, David Engblom, Celia Regina Cavichiolo Franco, Aleksander Roberto Zampronio

**Affiliations:** 1 Department of Pharmacology, Federal University of Paraná, Curitiba, PR Brazil; 2 Linköping University, Linköping, Sweden; 3 Federal University of Maranhão, São Luís, MA Brazil

**Keywords:** Cellular neuroscience, Molecular neuroscience

## Abstract

Lipopolysaccharide (LPS) induces fever through cytokines like receptor-activator of nuclear factor κB ligand (RANKL), triggering mediators like prostaglandins (PG), endothelin-1 (ET-1), corticotrophin-releasing factor (CRF), substance P (SP) and endogenous opioids. LPS-induced fever is reduced in females compared with males except in ovariectomized (OVX) females which show increased fever mediated by PG. The present study aimed to identify the mediators involved in fever in intact and OVX female rats. Fever was induced with LPS (50 μg/kg) intraperitoneally or CRF (2.5 μg), ET-1 (1 pg), morphine (10 μg) and SP (500 ng) intracerebroventricularly in sham-operated and OVX rats. The role of RANKL was evaluated with osteoprotegerin (OPG, 1 μg, intracerebroventricularly). Expression of RANK, CRF_I/II_, ET_B_, μ-opioid (MOR) and NK_1_ receptors was evaluated by confocal microscopy. Besides LPS, only morphine induced fever in OVX rats while all mediators induced fever in sham-operated animals. OPG abolished LPS-induced fever in OVX but not sham-operated animals. Overall, fever involves similar central mediators in cycling females and males but only morphine induced fever in OVX females. Importantly, RANK/RANKL participates in LPS-induced fever in OVX females, as in males but not in cycling females.

## Introduction

Fever is a symptom observed in numerous infectious and non-infectious diseases, classically defined as an elevation of bodily internal temperature above normal levels, caused by a shift on the hypothalamic thermoregulatory balance point^1^. Fever is a complex response, where signals originating in the periphery are integrated in the central nervous system and again promote peripheral effects, which are critical for defense against infections and stimulating host defense mechanisms^2,3^.

There are significant differences in the febrile response, inflammation and immunity between females and males^4–7^. The influence of sexual female hormones, and particularly estrogen, in these responses is documented^7^ even though more studies are necessary to reach a detailed comprehension of these influences. Most of these studies compare cycling females with males or cycling females with ovariectomized (OVX) females, the last one as a model of menopause, which may represent a different status in the pathophysiology related to female immunity.

Bacterial lipopolysaccharide LPS is a classic inducer of fever and inflammation, often used in experimental models to mimick the responses observed during natural infection. LPS stimulates Toll-like receptors (TLR) type 4, inducing the release of cytokines (often referred as endogenous pyrogens) like interleukin (IL)-1β, IL-1α, tumor necrosis factor-α (TNF-α), IL-6, IL-8, interferon β and γ and receptor-activator of NF-κB ligand (RANKL)^2,8–12^. While some of these, like IL-8 and RANKL may be directly generated in the central nervous system, others originate peripherally and act upon the CNS to trigger the febrile response^8,11–13^. When these cytokines reach the hypothalamic pre-optic area, they affect several relatively independent thermoeffector loops, triggering the release of other mediators – prostaglandins (PG), endothelin-1 (ET-1), corticotrophin-releasing factor (CRF), substance P (SP) and opioids - effectively changing the hypothalamic temperature set point, as reviewed by Zampronio *et al*.^14^.

Recently, our group also reported that female sex hormones influence the febrile response induced by LPS, PGE_2_, TNF-α and macrophage inflammatory protein (MIP)-1α, but not by IL-1β^7^. However, the mechanisms underlying the different susceptibility and responses of males and cycling and OVX females to pyrogenic stimuli remain largely obscure, partly because most of the works in this area are performed in male animals. A better understanding of these female-specific mechanisms is critical for developing more adequate therapies for women, especially in clinical settings involving inflammation-related pathologies (e.g. menopausal women).

A study conducted by Hanada *et al*.^8^, showed that RANKL also participated in the LPS-induced febrile response in male rats, as well as in physiological temperature regulation in female mice. The authors demonstrated that i.c.v. (but not i.p.) RANKL administration triggered severe fever in male mice and rats via COX-2 activation and PGE_2_ synthesis. The selective transgenic removal of RANK in neurons (Nestin-Cre) and and glial cells (GFAP-Cre) blocked the development of fever in response to LPS, IL-1β or TNF-α. Nestin-Cre and GFAP-Cre RANK (floxed) female mice also showed increased basal body temperatures compared with ovariectomized mice and wild-type controls, as reviewed by Hanada *et al*.^15^. The RANK/RANKL/OPG system is known for being involved in postmenopausal osteoporosis and controlling the onset of hormone-induced mammary cancer which makes the investigation of this system in the febrile response in females particularly important.

Taking this into account, the present work aims to explore the role of central nervous system mediators already identified in males such as CRF, SP, ET-1 and opioids and particularly RANKL on the LPS-induced febrile response in female rats, and the possible impact of ovariectomy on these responses.

## Results

### CRF increases body temperature in sham-operated but not OVX rats

**Figure 1:**
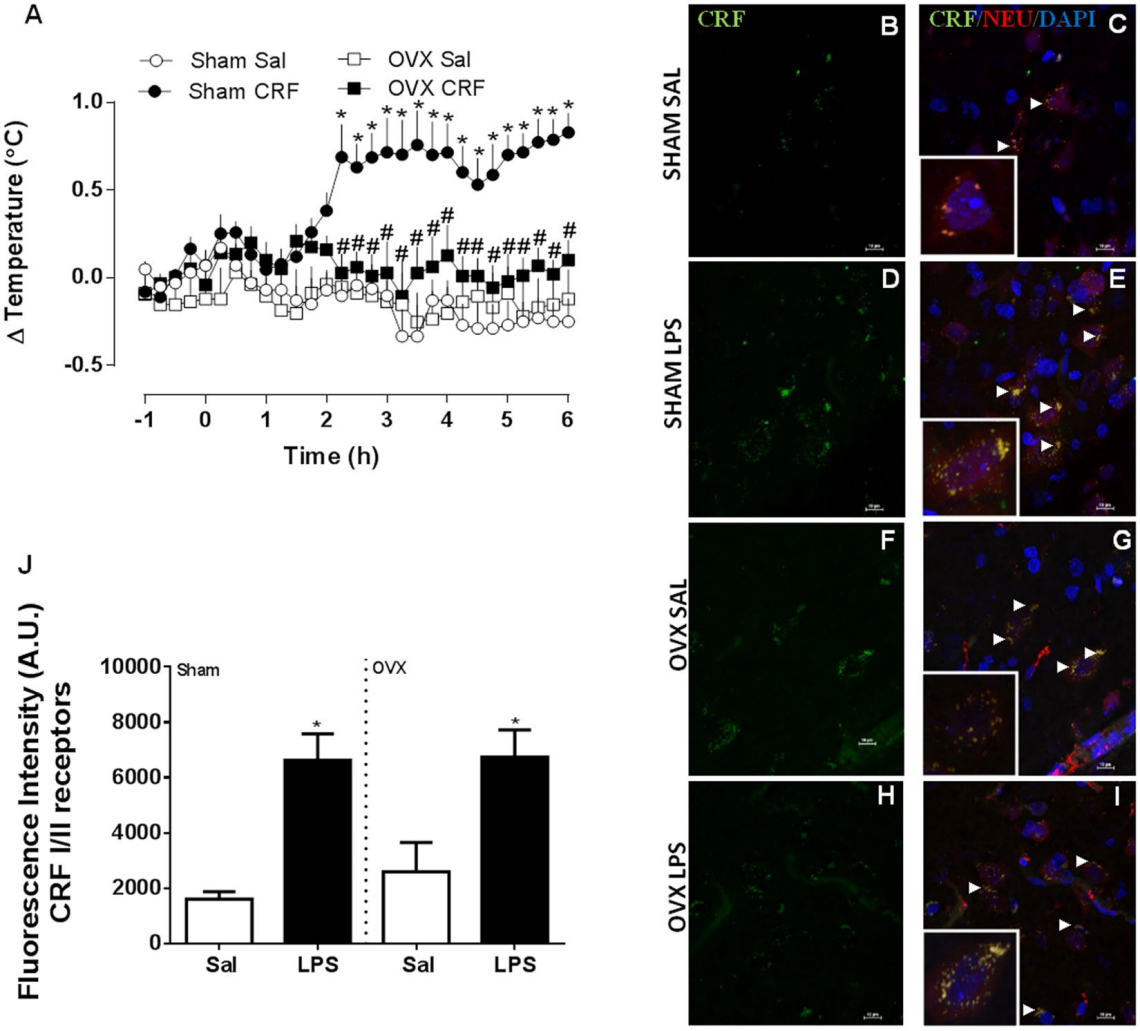
CRF induces a febrile response in sham-operated but not in ovariectomized (OVX) female rats. The animals were administered CRF (CRF, 2.5 µg/2 µL, i.c.v.) or saline (Sal). (**A**) mean ± s.e.mean of body temperature variation (°C) in each group (*n* = 4–7). *p < 0.05 compared with the saline group, ^#^*p* < 0.05 when compared with Sham CRF group. (**B–I**) Representative confocal images of hypothalamic samples from sham-operated OVX female rats, treated with LPS 50 µg/kg, i.p. or saline (100×). Groups are indicated at the left of each row. Panels B, D, F and H show immunoreactivity for CRF_I/II_ receptors (green), while panels C, E, G and I show merged images representing the green (CRF _I/II_), the blue (cell nuclei, DAPI), and the red (neurons, NEU) channels. Arrows point to co-localized signals (yellow). High-magnification inserts (400×) show co-localization in greater detail. (**J**) Fluorescence was quantified using ImageJ. Values are mean ± s.e.mean for CRF_I/II_ fluorescence intensity (arbitrary units, UA). *p < 0.05 when compared with the respective saline group.

Intracerebroventricular (i.c.v.) CRF administration in sham-operated rats induced a sharp temperature rise at 145 minutes, which peaked at 230 minutes and remained until the end of the experiment (Fig. 1A). In OVX rats, CRF administration failed to induce any temperature changes. Both sham-operated and OVX rats showed up-regulation of CRFI/II receptors when treated with LPS, which co-localized with hypothalamic neurons (Fig. 1B–J) but no difference was found between sham-operated and OVX animals.

### ET-1 increases body temperature in sham-operated but not OVX rats

**Figure 2:**
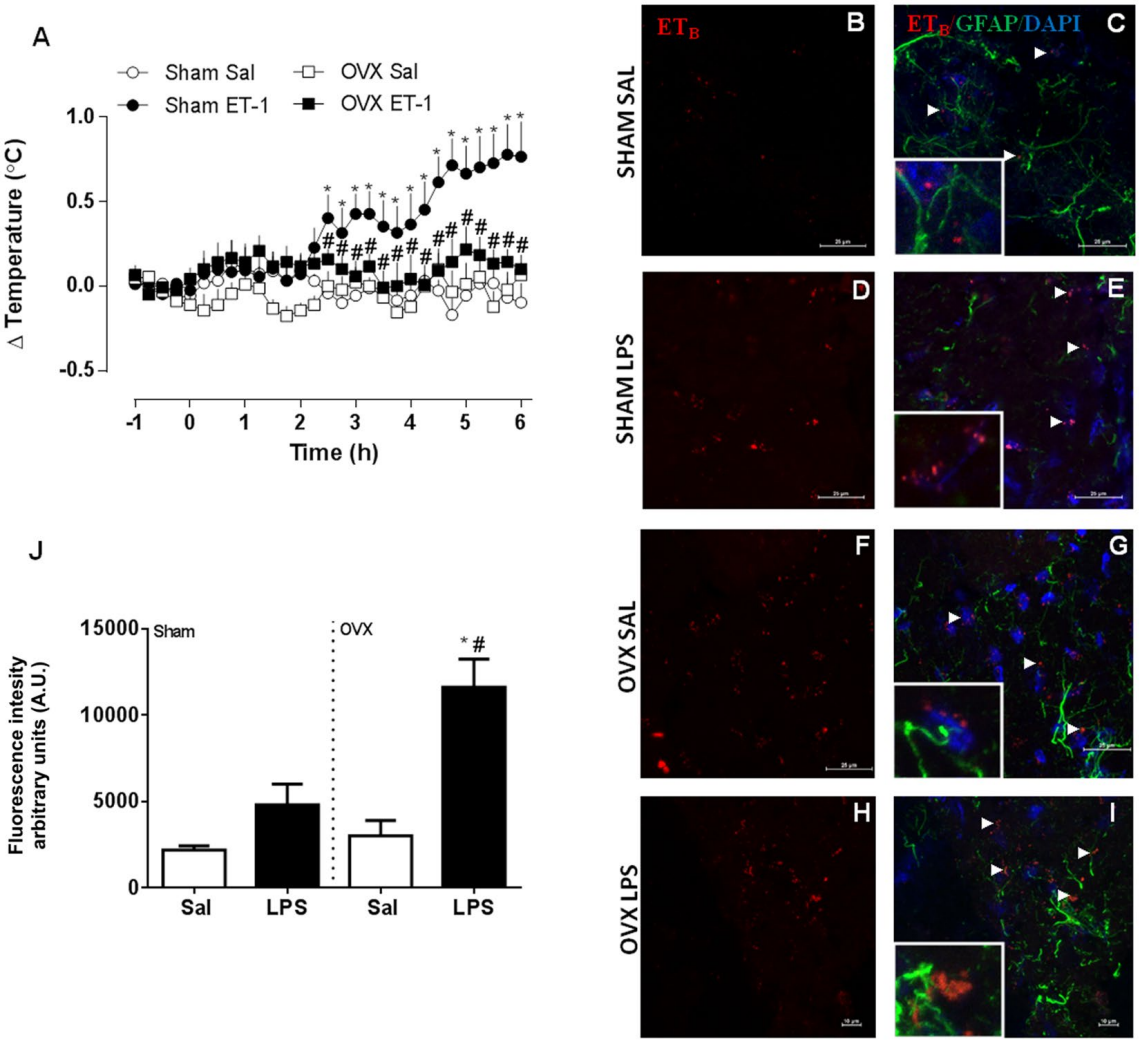
ET-1 induces a febrile response in sham-operated but not in ovariectomized (OVX) female rats. The animals were administered ET-1 (ET-1, 1 pg, i.c.v.) or saline (Sal). (**A**) mean ± s.e.mean of body temperature variation (°C) in each group (*n* = 4–7). *p < 0.05 compared with the saline group, ^#^*p* < 0.05 when compared with Sham ET-1 group. (**B–I**) Representative confocal images of hypothalamic samples from sham-operated and OVX female rats, treated with LPS 50 µg/kg, i.p. or saline (100×). Groups are indicated at the left of each row. Panels B, D, F and H show immunoreactivity for ET_B_ receptors (red), while panels C, E, G and I show merged images representing the red (ET_B_), the blue (cell nuclei, DAPI), and the green (astrocytes, GFAP) channels. Arrows point to ET_B_ signals. High-magnification inserts (400×) show localization in greater detail. (**J**) Fluorescence was quantified using ImageJ. Values are mean ± s.e.mean for ET_B_ fluorescence intensity (arbitrary units, UA). *p < 0.05 when compared with OVX Sal group; ^#^*p* < 0.05 when compared with Sham LPS group.

In the present study, i.c.v. ET-1 administration induced a temperature rise in sham-operated females, that started at 170 min post-administration, peaked at 315 minutes and remained until the end of the experiment (Fig. 2A). ET-1 had no effect on the body temperature of OVX female rats. LPS induced a slight increase in ET_B_ receptor expression in the hypothalamic neurons of sham-operated rats, which didn’t reach statistical significance (Fig. 2B–J). LPS-treated OVX rats showed significantly increased ET_B_ receptor expression, compared with all other experimental groups.

### Morphine increases body temperature in both sham-operated and OVX rats

**Figure 3:**
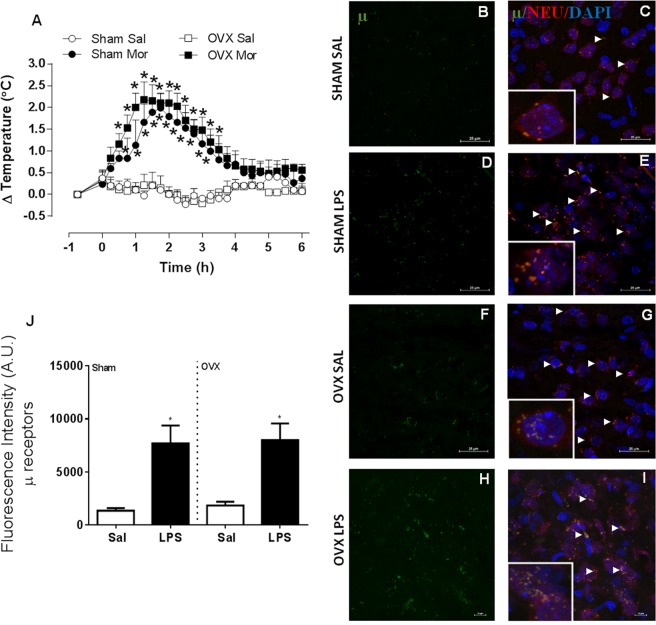
Morphine induces a febrile response in both sham-operated and ovariectomized (OVX) female rats. The animals were administered morphine (10 µg, i.c.v.) or saline (Sal). (**A**) mean ± s.e.mean of body temperature variation (°C) in each group (*n* = 4–7). *p < 0.05 compared with the saline group, #*p* < 0.05 when compared with the respective saline group. (**B–I**) Representative confocal images of hypothalamic samples from sham-operated and OVX female rats, treated with LPS 50 µg/kg, i.p. or saline (100×). Groups are indicated at the left of each row. Panels B, D, F and H show immunoreactivity for μ receptors (green), while panels C, E, G and I show merged images representing the green (μ receptors), the blue (cell nuclei, DAPI), and the red (neurons, NEU) channels. Arrows point to co-localized signals (yellow). High-magnification inserts (400×) show co-localization in greater detail. (**J**) Fluorescence was quantified using ImageJ. Values are average ± standard error for μ receptors fluorescence intensity (arbitrary units, UA). *p < 0.05 when compared with the respective Sal groups.

In sham-operated females, i.c.v. morphine administration induced a temperature rise that started at 45 min post-administration, peaked at 105 min and ended at 195 min (Fig. 3A). In OVX females, morphine also induced a temperature rise, which started at 30 min, peaked at 75 min and ended at 210 min. No significant differences were observed between sham-operated and OVX animals. Both OVX and sham-treated rats showed similar increased expression of µ receptors co-localizing with hypothalamic neurons, upon LPS administration (Fig. 3B–J).

### SP increases body temperature in sham-operated but not in OVX rats

**Figure 4:**
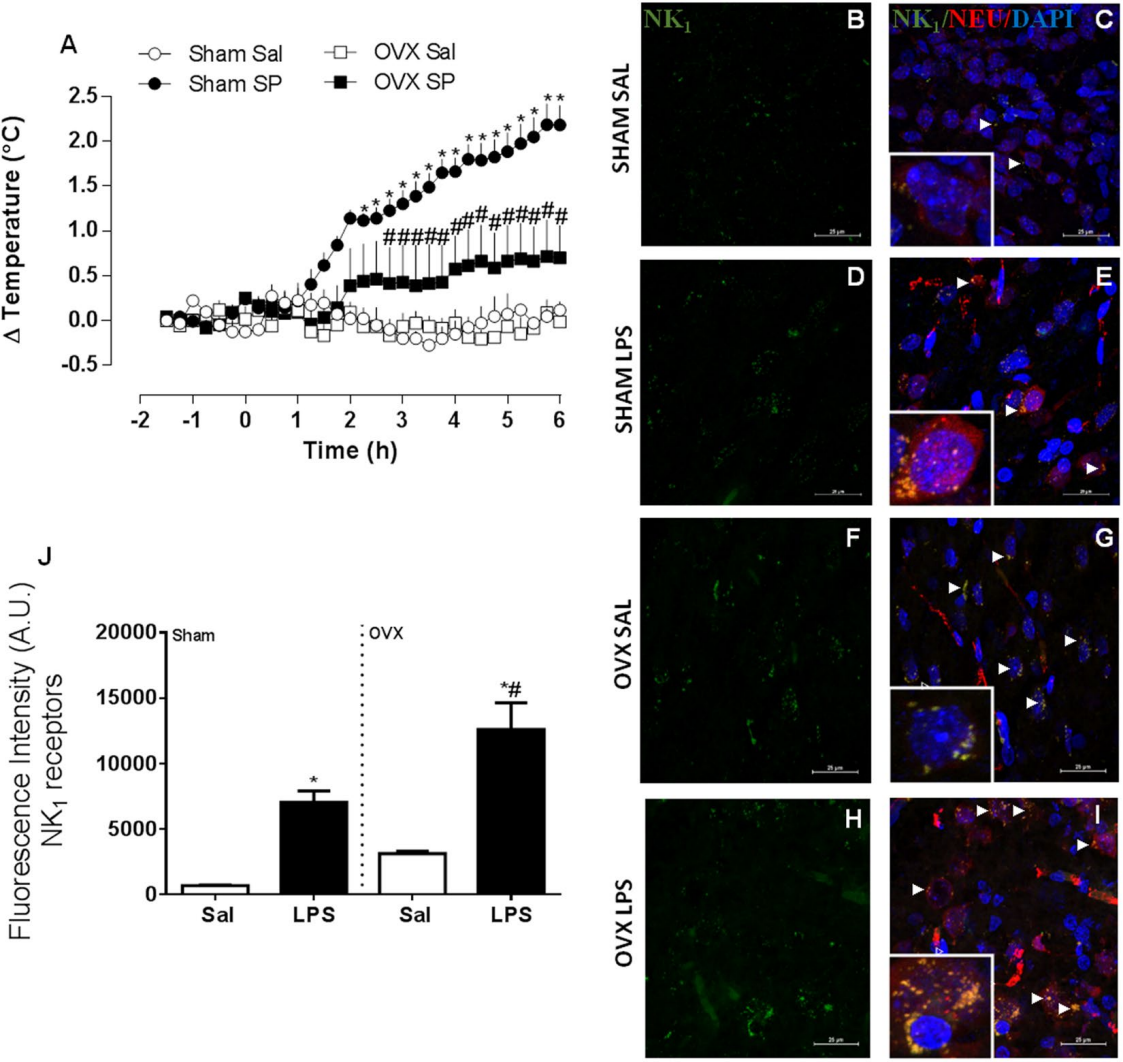
Substance P (SP) induces a febrile response in sham-operated and ovariectomized (OVX) female rats. The animals were pre-treated with captopril (5 µg/2 µl, i.c.v.) and SP (500 ng, i.c.v.) or saline (Sal) were administered after 30 min. (**A**) mean ± s.e.mean of body temperature variation (°C) in each group (*n* = 4–7). *p < 0.05 compared with the saline group, ^#^*p* < 0.05 when compared with the Sham SP group. (**B–I**) Representative confocal images of hypothalamic samples from sham-operated and OVX female rats, treated with LPS 50 µg/kg, i.p. or saline (100×). Groups are indicated at the left of each row. Panels B, D, F and H show immunoreactivity for NK_1_ receptors (green), while panels C, E, G and I show merged images representing the green (NK_1_), the blue (cell nuclei, DAPI), and the red (neurons, NEU) channels. Arrows point to co-localized signals (yellow). High-magnification inserts (400×) show co-localization in greater detail. (**J**) Fluorescence was quantified using ImageJ. Values are average ± standard error for NK_1_ fluorescence intensity (arbitrary units, UA). *p < 0.05 when compared with the respective Sal groups. ^#^*p* < 0.05 when compared with Sham LPS group.

We observed that i.c.v. SP administration induced a temperature rise in sham-operated females that started at 145 min post-administration and peaked at the end of the experimental period (Fig. 4A). No effects were observed in OVX rats. Both OVX and sham-treated rats showed increased expression of NK_1_ receptors upon LPS administration, which co-localized with hypothalamic neurons (Fig. 4B–J). This effect was more pronounced in LPS-treated OVX animals, which also formed clusters of NK_1_ receptors (Fig. 4P,Q).

### RANK antagonism reduces the febrile response in OVX females

**Figure 5:**
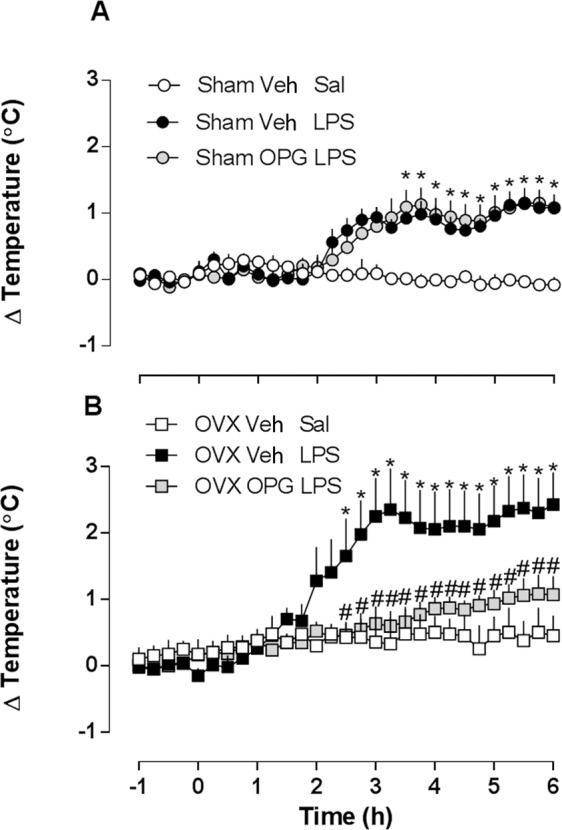
LPS-induced febrile response in sham-operated and ovariectomized (OVX) female rats pre-treated with RANK antagonist osteoprotegerin (OPG). Sham-operated (**A**) and OVX (**B**) female rats were pre-treated with OPG (1 µg/2 µl, i.c.v.) or saline (Sal, i.c.v.) 30 min before LPS or saline treatment (50 µg/kg, i.p.). Values are mean ± s.e.mean of body temperature variation (°C) in each group (*n* = 4–7). *p < 0.05 compared with the saline group, ^#^*p* < 0.05 when compared with OVX LPS females.

**Figure 6:**
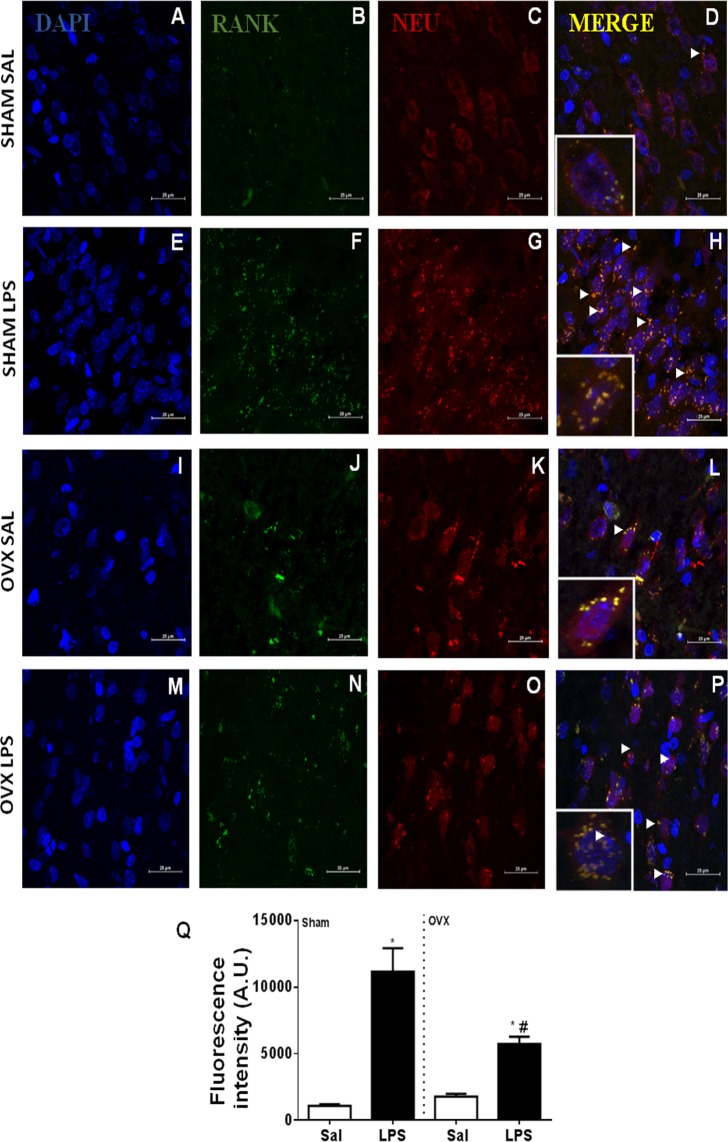
RANK receptors are up-regulated in hypothalamic neurons in LPS-treated rats. Representative confocal images of hypothalamic samples from sham-operated and ovariectomized (OVX) female rats, treated with LPS (50 µg/kg, i.p.) or saline (SAL) (100×). Groups are indicated at the left of each row. Panels A, E, I and M show the cell nucleus (DAPI, blue), B, F, J and N for RANK (green) and C, G, K and O for neurons (NEU, red), while panels D, H, L and P show merged images and co-localization of signals (yellow, arrows). High-magnification inserts (400×) show co-localization in greater detail. Q: fluorescence was quantified using ImageJ. Values are average ± standard error for RANK fluorescence intensity (arbitrary units, UA). *p < 0.05 when compared with Sham Sal and OVX Sal; ^#^*p* < 0.05 when compared with Sham Sal, Sham LPS and OVX Sal.

In order to study the involvement of RANK/RANKL in the febrile reaction to LPS in females, we administered the RANK decoy receptor osteoprotogerin (OPG) intracerebroventricularly (i.c.v.) at 1 µg/2 µL, 30 minutes before LPS administration. The treatment had no influence on the febrile response of sham-operated females (Fig. 5A), but significantly reduced the response of OVX rats (Fig. 5B). Next, we investigated the distribution of RANK receptors on the hypothalamus of sham-operated and OVX females, using confocal microscopy. Both groups showed RANK expression, which co-localized with neuronal bodies (Fig. 6A–P). RANK expression was up-regulated upon LPS administration in both sham-operated and OVX females, but this phenomenon was significantly more intense in sham-operated rats (Fig. 6Q).

### Distribution of receptors in neurons and astrocytes

**Figure 7:**
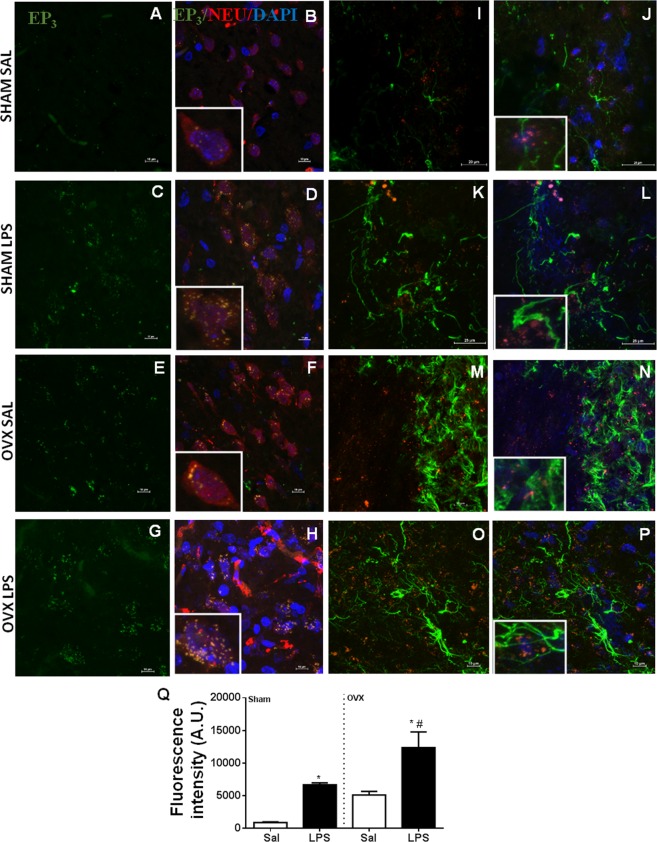
EP_3_ receptors are expressed in neurons but not astrocytes in LPS-treated rats. Representative confocal images of samples from sham-operated and OVX female rats, treated with LPS (50 µg/kg, i.p.) or saline (100×). Groups are indicated to the left of each row. Panels A, C, E and G show show immunoreactivity for EP_3_ receptors (green), (**B,D,F,H**) show merged images of blue (cell nuclei, DAPI), green (EP_3_), and red (neurons, NEU) channels. Panels I, K, M and O and M show double immunoreactivity for EP_3_ (red) and GFAP (astrocytes, green), (**J,L,N,P**) show merged images of blue (cell nuclei, DAPI), green (astrocytes, GFAP), and red (EP_3_) channels. Arrows point to EP_3_ imunoreactivity signals. Note co-localization with neurons but not astrocytes. High-magnification inserts (400×) highlight co-localization. Q: fluorescence was quantified using ImageJ (average ± standard error, arbitrary units, UA). *p < 0.05 compared with Sham Sal; ^#^*p* < 0.05 com*p*ared with OVX Sal.

We previously reported that OVX rats showed elevated hypothalamic levels of prostaglandin E_2_^7^. Here we studied the expression of the prostaglandin E_2_ receptor EP_3_ in hypothalamic samples of sham-operated and OVX females, using confocal microscopy (Fig. 7). LPS significantly increased the expression of EP_3_ receptors in sham-operated rats. Untreated OVX rats showed basal EP_3_ expression levels similar to those of LPS-treated sham-operated rats, and LPS treatment further increased EP_3_ expression (Fig. 7A–H and Q). The expression of this receptor was restricted to neurons, with no signals co-localizing with astrocytes (Fig. 7I–P).

**Figure 8:**
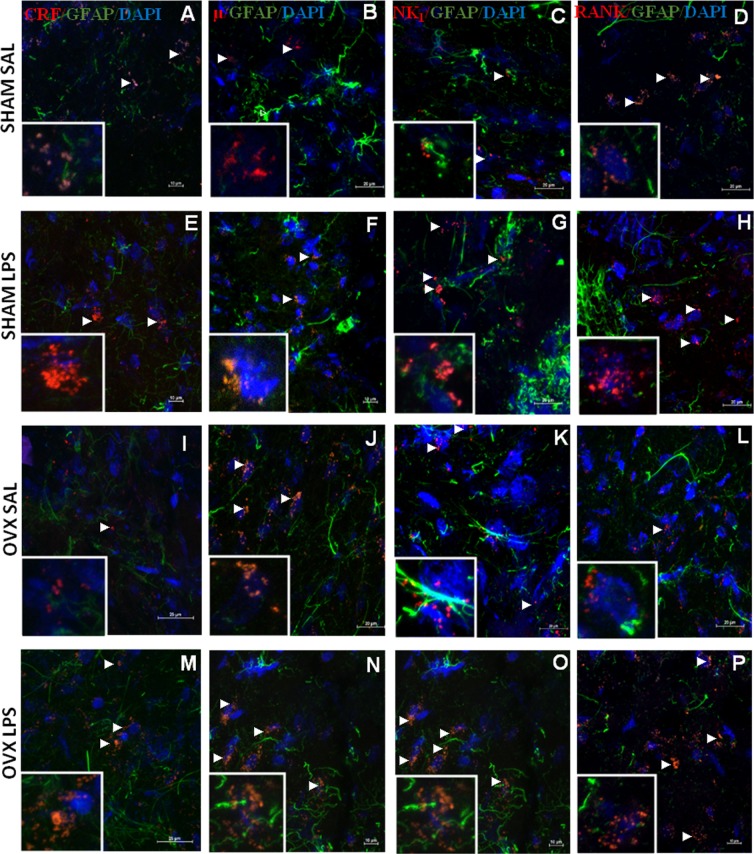
CRF_I/II_, μ, NK_1_ and RANK receptors are not expressed in hypothalamic astrocytes in LPS-treated rats. Representative confocal images of hypothalamic samples from sham-operated and ovariectomized (OVX) female rats, treated with LPS (50 µg/kg, i.p.) or saline (100×). All panels show merged images of blue (cell nuclei, DAPI), green (astrocytes, GFAP), and red (CRF_I/II_, μ, NK_1_ and RANK) channels. Panels A, E, I and M show immunoreactivity for CRF_I/II_, B, F, J and N for μ receptors, (**C,G,K,O**) for NK_1_. Arrows point to immunoreactivity signals of CRF_I/II_, μ, NK1 and RANK. Note absence of co-localization with astrocytes. High-magnification inserts (400×) show the lack of co-localization in greater detail.

Finally, we checked whether CRF_I/II_, μ, NK_1_ and RANK receptors were expressed in astrocytes using confocal microscopical analysis (Fig. 8). None of the signals for these receptors co-localized with signals for the astrocyte marker GFAP, suggesting their expression was restricted to neurons.

## Discussion

The present study shows that cycling sham-operated females display a febrile response like the one observed in males after the injection of some pyrogenic substances such as CRF, ET-1, morphine and SP. However, we show here that OVX females do not respond to most of these mediators (CRF, ET-1 and SP) but show a similar response to sham-operated females to morphine. Interestingly, the expression of EP_3_, ET_B_, and NK_1_ receptors was significantly increased in OVX females compared to sham-operated animals in the absence of pyrogenic stimulation by LPS. Also, sham-operated and OVX females show an increased expression of the receptors for these mediators in hypothalamic neurons in response to LPS. Additionally, RANKL participates in the febrile response induced by LPS in OVX females but not in cycling females which have a significantly increased expression of RANK receptors after LPS injection compared to OVX females in the same condition.

In a previous work^7^ we have shown that the production of prostaglandins in OVX female rats after LPS injection is controlled by female sex hormones. In the present study, we add new data regarding the febrile response in OVX females. We observed an increased expression of EP_3_ receptors in hypothalamic neurons of both sham-operated and OVX females after LPS injection and the expression of these receptors in OVX animals is higher than in sham-operated animals. No previous studies evaluated the expression of these receptors in the hypothalamus of female rats after ovariectomy. The involvement of hypothalamic EP_3_ receptors in fever was confirmed using EP_3_ knockout animals^16^. Additionally, Mouihate *et al*.^17^ showed that the expression of EP_3_ receptors is unchanged at near term pregnancy after LPS injection. We still do not know if these differences are sex-related or are due to the hormonal status analyzed (cycling versus near term pregnant versus OVX). However, our previous study^7^ showed that OVX female rats showed an increased febrile response to PGE_2_ i.c.v. injection which is consistent with the increased expression of EP_3_ receptors in the hypothalamus observed in the present study. Altogether, these results suggest that female sex hormones can modulate the prostaglandin system in females. While the presence of these hormones can downregulate COX-2 synthesis, their reduction can increase COX-2 expression and possibly EP_3_ expression, at least in the hypothalamus.

We next proceeded to investigate whether other central mediators of the febrile response^14^ might also be affected by female sex hormones. The febrile response induced by CRF in cycling sham-operated females was similar to the one observed in males^7,10^ suggesting that this peptide is also involved in fever in females. However, our results indicate that ovariectomy abolished the febrile response induced by CRF without changing the expression of CRF_I/II_ receptors. The response to IL-1β and some chemokines such as CCL3 and CXCL8 depends on CRF release^10,18,19^ and, in the case of IL-1β, is not affected by ovariectomy. Considering that ovariectomy increases the production and activity of prostaglandins, but reduced CRF, we speculate that the fever induced by IL-1β in OVX rats, which was unchanged in OVX female rats^7^ may rely more on prostaglandins and less on CRF, compared with males and with intact females.

The role of ET-1 and its ET_B_ receptor in febrile responses is well documented^20–22^. In males, this peptide seems to be produced dose-dependently in response to LPS^23^, and mediates LPS-induced fever, apparently through a prostaglandin-independent pathway^20,24^. Our results show that ET-1 induced a temperature increase in sham operated animals, and that this effect was abolished by ovariectomy, even though OVX females treated with LPS showed significantly increased ET_B_ expression, compared with all other groups. These findings suggest that ET-1 may be an important fever mediator in intact but not OVX females, and that this difference is not due to an absence in ET_B_ expression. Interestingly, the CRF-induced febrile response may be mediated by ET-1^25^, and thus seems to be highly influenced by female sex hormones. The increased expression of ET_B_ receptors in the hypothalamus may indicate a direct regulation of sex hormones on the expression of these receptors or a reduction in the production of ET-1 in OVX animals. Gohar *et al*.^26^ also showed that ovariectomy increases the expression of ET_B_ receptors in the heart and renal inner medulla, but not in the lungs, which was normalized after estrogen replacement. Previous studies show that OVX rats have increased levels of plasma ET-1, preproET-1 mRNA and endothelin-converting enzyme and estrogen administration reduced these parameters^27^. Nevertheless, in a recent study, Subramanian *et al*.^28^ showed that the chronic exposure to low doses of estradiol in female rats increased the expression of ET-1 mRNA transcripts in the paraventricular nucleus and the rostral ventrolateral medulla, suggesting that an increased central production of ET-1 is under control of these hormones. So it is plausible to think that ovariectomy would reduce the production of central ET-1 which might lead to an increase expression of endothelin receptors. Hormonal reposition experiments in OVX females will be necessary to confirm this hypothesis.

The role of opioids in the febrile response triggered by LPS, as well as by several mediators like TNF-α, IL-6, CCL3, PGF_2α_, CRF e ET-1 has been well studied in males^29–32^. In the present study, morphine induced a similar febrile response in sham-operated and OVX females. Female sex hormones did not exert any effects on the morphine-induced febrile response nor on µ receptor expression, suggesting that hormonal regulation may occur at an upstream level. Considering that the fever induced by CCL3, CRF and ET-1 all depend on endogenous opioids^32^, we speculate that female sex hormones may regulate the febrile response mainly at those levels.

Substance P is a less studied fever mediator, but its participation in the febrile response is considered crucial^7,33^. In our study, substance P increased body temperature in sham-operated animals but not in OVX rats. While LPS increased the expression of NK_1_ receptors in both groups, the effect was much more pronounced in OVX rats, which also showed clustering of the receptor. Considering these results, it is possible that ovariectomy abolished the fever induced by TNF-α (contrary to IL-1β) because that mediator (but not IL-1β) acts through substance P and the activation of NK_1_ receptors^7^. On the other hand, it is also possible that OVX females require a higher dose of ACE inhibitors to allow SP activity compared with intact females and this interesting point should be clarified by additional studies. In their study, Hanada *et al*.^8^ showed that RANKL is an important mediator of fever induced by LPS, IL-1β and TNF-α in male mice, and that it acts by recruiting prostaglandins. The authors didn’t perform pharmacological treatments in females, but reported that Nestin-Cre and GFAP-Cre rank (floxed) female mice showed increased basal body temperatures compared with ovariectomized mice and wild-type controls, suggesting a female hormonal influence. It is important to emphasize that RANK expression in both neurons and astrocytes seemed to be important for temperature regulation in females. In the present study, we observed that, in contrast with males^8^ the febrile response induced by LPS in sham-operated females was not reduced by OPG suggesting that RANKL is not involved in fever in cycling females. However, we observed that OVX females treated with a single dose of the RANKL decoy-receptor OPG showed a reduced febrile response, compared with untreated OVX animals. These results suggest that the involvement of RANK/RANKL/OPG system in the febrile response is prominently influenced by female sex hormones. RANK expression induced by LPS was higher in neurons of sham-operated animals compared with OVX, suggesting that a down-regulation of the receptor for female hormones is not the reason why RANKL does not participate in the febrile response in cycling animals. These results suggest that the RANK/RANKL/OPG system is influenced by female hormones and plays an important role in fever induction in females only when hormonal levels are low, such as during menopause.

Among all the receptors we studied (EP_3_, CRF_I/II_, ET_B_, µ, NK_1_ and RANK), none co-localized with astrocytes, but only with neurons, regardless of the treatment. These data are in contrast with previous studies showing the presence of CRF _I/II_, NK_1_ e ET_B_ in both neurons and astrocytes^34–37^. Opioid µ receptors have been detected in astrocytes, neurons, microglial cells and the endothelium^38^ and *in vitro* studies suggest that morphine alters the function of microglial cells and astrocytes^39^. There are less data concerning RANK, but Hanada *et al*.^8^ showed it is expressed in astrocytes.

OVX females, differently from males, seem to have a febrile response dependent on an increased production of prostaglandins (previous results) and endogenous opioids but not other central mediators such as CRH, ET-1, or substance P. Additionally, RANK/RANKL seems to play a major role in the LPS-induced febrile response in OVX females but not in cycling females. The expression of RANK, CRF_I/II_, EP_3_, ET_B_, µ and NK_1_ receptors was significantly increased in intact females following LPS administration, while OVX rats only showed EP_3_, ET_B_ and NK_1_ receptors up-regulation. A better understanding of these female-specific mechanisms is critical for developing more adequate therapies for women, especially in clinical settings involving inflammation-related pathologies (e.g. menopausal women).

## Methods

### Animals

Experiments were conducted in female Wistar rats (180–220 g), housed four per cage at 22 ± 1 °C, under a 12:12-h light-dark cycle (lights on at 0700 AM) and with free access to food chow and tap water. Animals were used only once. All experiments were approved by the Federal University of Paraná’s Ethical Committee in Animal Use and were performed in accordance with Brazilian and International animal welfare legislation.

### Drugs

*Escherichia coli* lipopolysaccharide (0111:B4), CRF, ET-1, morphine, and substance P abd captopril were all purchased from Sigma Chemical & Co., St. Louis, U.S.A. Human OPG was purchased from Peprotech Inc, Ribeirão Preto, Brazil. Anesthetics were ketamine (Vetnil Veterinary Products, Louveira, Brazil) and xylazine (Syntec Laboratory, Cotia, Brazil). Antibiotic was oxytetracycline hydrochloride (Pfizer Laboratories, São Paulo, Brazil).

### Experimental design and statistical analysis

Abdominal core temperature (Tc) was evaluated for 6 h. All pyrogenic stimuli were injected between 09:00 and 11:00 AM to avoid possible circadian variations in body temperature. Procedures and the doses of pyrogenic stimulus were based on previous studies^32^. The animals were pre-treated with captopril (5 µg/2 µl, i.c.v.) 30 minutes before administering substance P. Sample size for each group is reported in the figure legends. Temperature recording were analyzed by two-way ANOVA for repeated measures followed by Bonferroni’s test for *post hoc* analysis. Confocal microscopy experiments were performed with 3 to 4 animals in each group and the analysis was performed with at least 6 to 10 cuts per animal. Mean intensities in confocal quantification were analyzed by two-way ANOVA followed by Bonferroni’s test. Results are reported as means ± s.e.m. All data were analyzed using GraphPad Prism 6 software (GraphPad, San Diego, USA). differences in experiments were considered significant when *P* values were <0.05.

### Ovariectomy

Ovariectomy was performed as previously described^40^. Briefly, female rats were anesthetized with ketamine (90 mg/kg) and xylazine (7.5 mg/kg) i.p. and under aseptic conditions a laparotomy of 2 cm was done in the median line. The ovaries and the fallopian tubes were separated. Fallopian tubes were ligated with sutures and the ovaries removed. The abdominal cavity was sutured and anestrous was confirmed after 21 days. Sham-operated animals were submitted to the same procedure, but ovaries and tubes were kept intact.

### Intracerebral cannula implantation and microinjection

Intracerebroventricular (i.c.v.) administrations were performed as previously described^40^. Briefly, a 22-gauge stainless steel guide cannula (0.8 mm outer diameter, 12 mm long) was stereotaxically implanted into the right lateral ventricle under ketamine (90 mg/kg) and xylazine (7.5 mg/kg) anaesthesia in aseptic conditions. The stereotaxic coordinates were 0.8 mm lateral to the midline, 1.5 mm posterior to the bregma, and 2.5 mm below the brain surface, with the incisor bar lowered by 3.3 mm below the horizontal zero^41^. Cannulas were fixed with jeweller’s screws embedded in dental acrylic cement to the skull. Animals received oxytetracycline hydrochloride (400 mg/kg, by intramuscular route) after surgery and were allowed to recover for at least 5 days before the experiment. After the experiment, animals were injected into the lateral ventricle with Evans blue (2.5% in saline). Brains were removed and the animals showing cannula misplacement, blockage on injection, or abnormal body weight gain patterns after surgery were excluded from the study.

### Temperature recordings

Tc was measured as previously described^40^ in conscious unrestrained rats using data loggers (Subcue, Calgary, Canada). Briefly, data loggers were implanted in the peritoneal cavity of OVX or sham-operated rats, at least 5 days before the experiment, under the same anaesthesia described before and received the same post-surgical care. On the day of the experiment, Tc was continuously monitored at 15-min intervals from 2 h before any injection until 6 h after the injection of the pyrogenic stimulus. During the experiment, room temperature was maintained at 28 ± 1 °C (within the thermoneutral zone for rats)^42^. Animals displaying basal temperatures below 36.7 °C or above 37.4 °C were excluded from the study.

### Confocal Immunofluorescence protocols

Three hours after the LPS/saline administrations the brains were removed for analysis of EP_3_, RANK, CRF_I/II_, ET_B_, μ-opioid or NK_1_ receptor expression in the anterior hypothalamus, identifying their co-expression in astrocytes and neurons. For this, sham-operated animals and OVX were treated with LPS (50 μg/kg, i.p) or an equi-volume of saline solution. The animals were anesthetized with ketamine/xylazine and were perfused via transcardiac incision through the left ventricle with 300 ml of PBS, 0.1 mM, pH 7.4. This was followed by 4% paraformaldehyde (PFA), except for animals meant for ET_B_ evaluation. Following their removal from the cranial cavity, the brains were cryoprotected through immersion in increasing concentrations of sucrose (10%, 20%, and 30%). The samples were soaked in Tissue-Tek® and rapidly frozen in liquid nitrogen. Brain slices were sectioned to a thickness of 30 μm in serial sections on Leica CM3050 cryostat (Leica Microsystems, Singapore) according to rat brain coordinates to separate the hypothalamic tissue^41^. All immunofluorescence reactions were performed using the free-floating method. Initially, the sections were washed 4 times with PBS (pH 7.4) to remove impurities from the cut processing, followed by a wash with 0.3% Triton X-100 for 1 min. Subsequently, the samples were incubated with 1% glycine solution for 5 min followed by blocking solution with 1% bovine serum albumin solution (BSA) for 1 h. Double immunostaining techniques were performed to identify neurons and astrocytes and the various receptors under study: CRF _I/II_, NK_1_, μ-opioid, EP_3_, RANK and ET_B_.

The sections were incubated overnight with the following primary antibodies: anti-NeuN (MAB377, mouse, 1: 500, Millipore, Massachusetts, USA), for neurons, or anti-GFAP (sc-51908, mouse, 1:100, Santa Cruz Biotechnology, Inc., Dallas, USA) for astrocytes. Sections were then washed with 0.3% Triton X-100 blocking solution (3×/5 min) followed by PBS (2×/2 min). Then the sections were incubated with primary antibodies for the following markers: CRF _I/II_ (1: 50, sc-5543, rabbit), NK_1_ (1: 50, sc-14115, rabbit), EP_3_ (1: 50, sc-20676, rabbit), RANK (1: 300, sc-9072, rabbit) and ET_B_ (1: 50; sc-33538, rabbit) all from Santa Cruz Biotechnology, Inc., Dallas, USA for 12 h. Subsequently, the sections were washed with 0.3% Triton X-100 (3×/5 min) followed by PBS solution (2×/2 min). The following secondary antibodies were used: Texas red-conjugated anti-mouse (T862, 1: 300, ThermoFisher Scientific, Waltham, USA) for neurons or FITC-conjugated anti-mouse (A11063, ThermoFisher Scientific, Waltham, USA) for astrocytes for 2 h. Thereafter sections were washed again with 0.3% Triton X-100 (3×/5 min) followed by PBS (2×/2 min). Secondary antibodies to detect expression of receptors were FITC-conjugated anti-rabbit (F-0382, Sigma Aldrich & Co., USA, for double labeling with neurons) or Alexa Fluor 647-conjugated anti-rabbit (A31573, ThermoFisher Scientific, Waltham, USA, for double labeling with astrocytes). The reaction was terminated with sequential washes of Triton-X100 and PBS as above.

Controls were included as follows: 1- blank group with DAPI but no primary or secondary antibodies and 2- secondary control group, with the secondary antibody and DAPI in the absence of each primary antibody. The sections were mounted using Fluoromont-G staining medium with DAPI (# 17984-24, Eletron Microscopy Sciences, Hatfield, U.S.A.) for nuclear visualization and observed under a Nikon A1RiMP inverted fluorescence microscope (Nikon, Tokyo, Japan), at 60X and 100X in oil. The images (optical images of Z) were visualized and analyzed using Nis Elements 4.20 (NIKON, Tokyo, Japan). The fields for analysis were randomly chosen within the hypothalamus and their fluorescence intensity was quantified using ImageJ 1.4 (National Institutes of Health, Maryland, U.S.A.). Results were expressed in arbitrary units.

## Data Availability

All data generated or analyzed during this study are available from the corresponding author on reasonable request.
